# Unexpected diagnosis of myotonic dystrophy type 2 repeat expansion by genome sequencing

**DOI:** 10.1038/s41431-022-01166-y

**Published:** 2022-08-09

**Authors:** Haloom Rafehi, Cherie Green, Kiymet Bozaoglu, Greta Gillies, Martin B. Delatycki, Paul J. Lockhart, Ingrid E. Scheffer, Melanie Bahlo

**Affiliations:** 1 The Walter and Eliza Hall Institute of Medical Research, Melbourne, VIC Australia; 2 Department of Medical Biology, University of Melbourne, Melbourne, VIC Australia; 3 Department of Psychology and Counselling, School of Psychology and Public Health, La Trobe University, Melbourne, VIC Australia; 4 Bruce Lefroy Centre for Genetic Health Research, Murdoch Children’s Research Institute, Melbourne, VIC Australia; 5 Department of Medicine, The University of Melbourne, Austin Health, Melbourne, VIC Australia; 6 Department of Paediatrics, The University of Melbourne, Melbourne, VIC Australia; 7 Victorian Clinical Genetics Services, Melbourne, VIC Australia; 8 Florey Institute, Melbourne, VIC Australia; 9 Murdoch Children’s Research Institute, Melbourne, VIC Australia

**Keywords:** Movement disorders, Medical genomics

## Abstract

Several neurological disorders, such as myotonic dystrophy are caused by expansions of short tandem repeats (STRs) which can be difficult to detect by molecular tools. Methodological advances have made repeat expansion (RE) detection with whole genome sequencing (WGS) feasible. We recruited a multi-generational family (family A) ascertained for genetic studies of autism spectrum disorder. WGS was performed on seven children from four nuclear families from family A and analyzed for REs of STRs known to cause neurological disorders. We detected an expansion of a heterozygous intronic CCTG STR in *CNBP* in two siblings. This STR causes myotonic dystrophy type 2 (DM2). The expansion did not segregate with the ASD phenotype. Repeat-primed PCR showed that the DM2 CCTG motif was expanded above the pathogenic threshold in both children and their mother. On subsequent examination, the mother had mild features of DM2. We show that screening of STRs in WGS datasets has diagnostic utility, both in the clinical and research domain, with potential management and genetic counseling implications.

## Introduction

The advent of Whole Exome and Whole Genome Sequencing (WES/WGS) has been instrumental in the rapid genetic diagnosis of many conditions, including neurological disorders in both clinical and research settings. More recently, there have been significant advances in the development of bioinformatic methods for the detection of repeat expansions (REs) of short tandem repeats (STRs) in short-read sequencing datasets, such as exSTRa and ExpansionHunter [1]. These tools have proven successful for the diagnosis of conditions caused by known REs, and in the discovery of new REs [2, 3]. In this study, we recruited a multi-generational family (Family A) with multiple incidences of autism spectrum disorder (ASD), in order to study generic determinates of ASD, including REs. Fragile X syndrome is caused by a RE in *FMR1* has a 50% incidence of ASD co-morbidity [4]. ASD has also been reported as a symptom of myotonic dystrophy type 1 (DM1), a rare genetic disorder caused by a RE in *DMPK*. Here we show that applying a standard RE screening pipeline to Family A resulted in an unexpected diagnosis of myotonic dystrophy type 2 (DM2), caused by a CCTG repeat expansion in *CNBP* [5], in a sub-branch of the family.

## Methods

We recruited an extended multi-generational family (Family A) ascertained for genetic studies of ASD (Royal Children’s Hospital ethics approval #25043, with written informed consent). Genomic DNA was isolated from saliva. Paired-end 150 bp WGS was performed on seven children, siblings or first-degree cousins, from four nuclear families from within Family A using TruSeq Nano Library Preparation Kit and the Illumina HiSeq X Ten platform. Alignment was performed based on the GATK best practice pipeline. Fastq files aligned to the hg19 reference genome using BWA-mem, then duplicate marking, local realignment, and recalibration were performed with GATK and analyzed for REs of STRs known to cause neurological disorders using RE detection tools exSTRa [6] and ExpansionHunter [7], as previously described [2]. A database of pre-defined pathogenic REs was used: https://github.com/bahlolab/exSTRa/blob/master/inst/extdata/repeat_expansion_disorders_hg19.txt (file version committed on Nov 21, 2019).

These tools utilize paired-end reads to detect expanded STRs: exSTRa uses an empirical Cumulative Distribution Function (ECDF) to determine outliers, while ExpansionHunter estimates the number of repeats in the STR on each allele. Repeat-primed PCR [8] was used to test for expansion of the RE and was performed by PathWest Laboratory Medicine WA. Twenty to 200 ng of DNA were amplified in a reaction volume of 30 μl, using 1.5 units of GoTaq Polymerase (Promega), supplied buffer 1×, MgCl2 1.25 mmol/L, DMSO 10%, 7-DeAZA-GTP (Roche) 0.2 mmol/L, primers P1 (Fam-5′-GCCTAGGGGACAAAGTGAGA-3′) 3 pmol, P4 (5′-TACGCATCCGAGTTTGAGACGCCTGCCTGCCTGCCTGCCTG-3′) 0.6 pmol, P3 (5′-TACGCATCCCAGTTTGAGACG-3′) 3 pmol, with the following cycling conditions: initial denaturation 5 min/94 °C, followed by 35 cycles of (30 s/94 °C; 30 s/56 °C; 2 min/72 °C) and final extension of 10 min/72 °C, then stored at + 4 °C until analyzed. In the prospective study, every analysis included at least one DM2-positive and one DM2-negative sample as controls of PCR reaction.

## Results

**Fig. 1:**
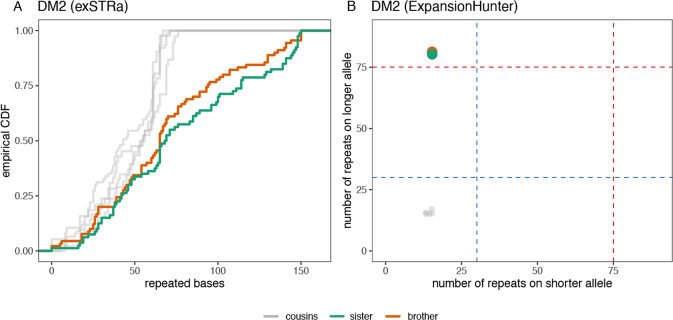
Detection of a DM2 repeat expansion in two siblings using WGS. The DM2 repeat expansion was detected in two siblings (green and orange) using the tools (a) exSTRa (shown as ECDF: Empirical Cumulative Distribution Function) and (b) ExpansionHunter (red dotted line indicates pathogenic threshold of 75 repeats, blue dotted line: benign/stable STR threshold of 30 repeats), compared with their first cousins who do not carry the allele, shown in gray. The ECDF plots the number of base pairs with in each read at the locus that are comprised of the repeated motif, sorted by size. A shift to the right on the plot in a sample compared to other samples indicates a potential RE.

**Fig. 2:**
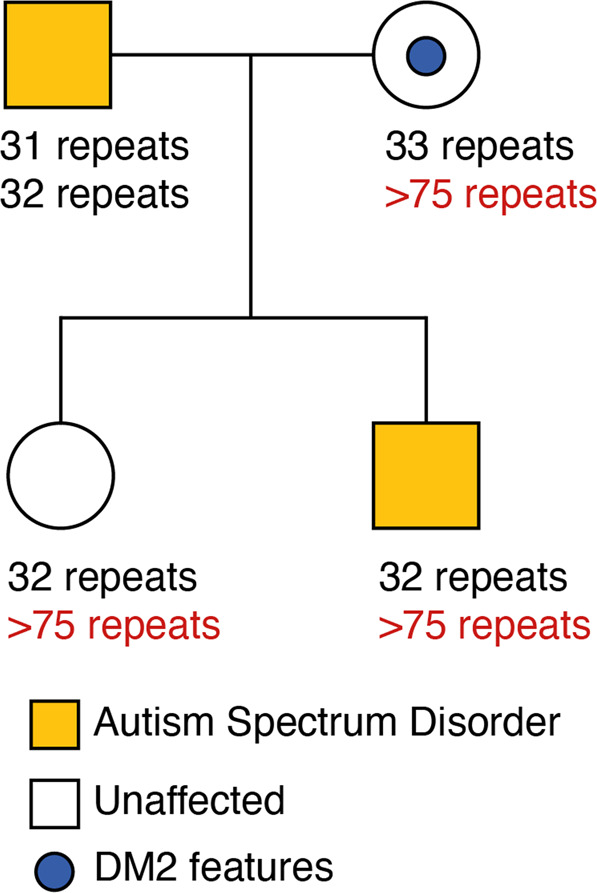
Pedigree showing affected status for ASD (yellow). The estimated number of base pairs in the CCTG STR in *CNBP* on each allele is shown for each family member, noting that the father does not carry an expanded allele. The expanded alleles are highlighted in red. 300 base pairs corresponds to 75 repeat units, which is the pathogenic threshold for DM2.

We detected an expansion of a heterozygous intronic CCTG STR in the gene *CNBP*, which is known to cause DM2, in a brother and sister pair (Fig. 1). Results from exSTRa (Fig. 1a) identify excess reads containing the CCTG motif in the two siblings compared to their cousins, indicating an expanded allele. ExpansionHunter (Fig. 1b) was used to estimate the allele repeat to be approximately 80 repeats in size in both siblings, which is larger than the 75 repeat threshold for pathogenicity for DM2. Although this estimated RE size is small compared to the very large expansions of up to 10,000 repeats that have been reported in DM2, it is well established that in silico tools do not provide accurate estimates for larger expansions [9]. Repeat-primed PCR was performed and confirmed that the DM2 CCTG motif was expanded above the pathogenic threshold (>75 repeats) in both children. Their parent was also found to have a CCTG expansion (>75 repeats) in *CNBP* (Fig. 2). On follow-up examination, the affected parent had mild features of DM2, including the classical finding of percussion myotonia [10]. This DM2 RE was not detected in any of their cousins in the broader family, regardless of ASD status, and notably did not segregate with their father who had ASD.

## Discussion

Here, through the implementation of an advanced bioinformatic pipeline, we have made an unexpected incidental diagnosis of DM2 in three related individuals. Our pipeline incorporated enhanced RE detection methods to increase the diagnostic utility of WGS in neurological disorders. DM2 is typically a late-onset condition. In contrast to myotonic dystrophy type 1 (DM1), it has a relatively mild phenotype and anticipation is rarely observed. The subtle features identified on reviewing the family, evident only in the mildly affected mother, suggest that the diagnosis of DM2 can be missed due to its relatively mild phenotype. Unlike DM1 [11], DM2 has not been associated with ASD, suggesting that DM2 is an incidental finding in this family. This is further supported by the clinical genetic pattern of inheritance of ASD which extends through the paternal, rather than the maternal, line.

The missed diagnosis of DM2 in this family, despite clinical symptoms in the affected mother, highlights that repeat expansions disorders remain underdiagnosed. A molecular diagnosis is important for optimal management of the symptoms of DM2 and for genetic counseling [4]. Critically, we identified this repeat expansion in a family that was recruited for ASD research, highlighting the utility of this pipeline for the diagnosis of incidental neurological disorders in cohorts not thought to have REs.

Although this RE represents a likely incidental finding, we originally pursued the discovery due to the potential association of DM1 and ASD [11], speculating that the even less common and more subtle features of DM2 might be a hidden contributor to ASD likelihood. However the DM2 RE did not segregate with ASD in this family. Furthermore, a recent screen of children with ASD (*N* = 1812) identified DM1 expansion in seven individuals with ASD (OR = 1.37), no expansions were identified in DM2, providing additional evidence that DM2 is not associated with ASD [12].

The use of bioinformatic tools to identify REs in WGS datasets has not been broadly applied in research and clinical settings. Indeed, recent publications still incorrectly state that WGS cannot be used to detect RE [13], despite multiple demonstrations of their utility across a wide range of RE disorders. We show that the implementation of an STR analysis pipeline to screen all WGS datasets has considerable diagnostic utility, both in the clinical and research domain.

## Data Availability

The datasets generated during and/or analyzed during the current study are not publicly available due to specific ethics that does not allow public sharing of genomic data, but are available from the corresponding author on reasonable request.
